# Highly Stretchable and Self-Healing Strain Sensors Based on Nanocellulose-Supported Graphene Dispersed in Electro-Conductive Hydrogels

**DOI:** 10.3390/nano9070937

**Published:** 2019-06-28

**Authors:** Chunxiao Zheng, Yiying Yue, Lu Gan, Xinwu Xu, Changtong Mei, Jingquan Han

**Affiliations:** 1 College of Materials Science and Engineering, Nanjing Forestry University, Nanjing 210037, China; 2 College of Biology and Environment, Nanjing Forestry University, Nanjing 210037, China

**Keywords:** nanocellulose, poly (vinyl alcohol), graphene, borax, hydrogel, self-healing, conductive, stretchable, viscoelasticity, strain sensor

## Abstract

Intrinsic self-healing and highly stretchable electro-conductive hydrogels demonstrate wide-ranging utilization in intelligent electronic skin. Herein, we propose a new class of strain sensors prepared by cellulose nanofibers (CNFs) and graphene (GN) co-incorporated poly (vinyl alcohol)-borax (GN-CNF@PVA) hydrogel. The borax can reversibly and dynamically associate with poly (vinyl alcohol) (PVA) and GN-CNF nanocomplexes as a cross-linking agent, providing a tough and flexible network with the hydrogels. CNFs act as a bio-template and dispersant to support GN to create homogeneous GN-CNF aqueous dispersion, endowing the GN-CNF@PVA gels with promoted mechanical flexibility, strength and good conductivity. The resulting composite gels have high stretchability (break-up elongation up to 1000%), excellent viscoelasticity (storage modulus up to 3.7 kPa), rapid self-healing ability (20 s) and high healing efficiency (97.7 ± 1.2%). Due to effective electric pathways provided by GN-CNF nanocomplexes, the strain sensors integrated by GN-CNF@PVA hydrogel with good responsiveness, stability and repeatability can efficiently identify and monitor the various human motions with the gauge factor (GF) of about 3.8, showing promising applications in the field of wearable sensing devices.

## 1. Introduction

Recently, flexible, stretchable, self-healing and human-friendly devices have gained widespread attention for multi-functional wearable electronics [1,2]. As the essential components in soft electronics, the stretchable strain sensors that can perceive human movement have promoted rapid development of personalized health detection, human motion monitoring, E-skins and robots [3,4,5,6,7,8,9]. Hydrogels with a 3D polymer network can preserve large amounts of water. During deformation, these soft materials can maintain their structural integrity and exhibit intrinsic flexibility, stretchability and even self-healing ability, which are considered as a suitable candidate for the fabrication of strain sensors [2]. Currently, electro-conductive hydrogels (ECHs) are emerging as a promising class of functional and intelligent soft materials because of their synergic electro-conductibility, rheological viscoelasticity and inherent flexibility [10]. ECHs generally consist of two parts, a conductive polymer and a hydrogel matrix, and each has its own unique properties. The conductive polymers are embedded in the hydrogel network via copolymerization, grafting or cross-linked reaction, which can retain the excellent properties of the hydrogel while endowing the electron transport capability with hydrogels. However, most ECHs currently suffer from weak mechanical strength, insufficient viscoelasticity, lack of self-healing ability and poor electrical conductivity, which can hardly satisfy the demands of practical applications.

The introduction of nanocellulose into hydrogel matrixes is an important method to improve their mechanical properties while achieving functionalization. Nanocellulose with cellulose backbone chains inherently forms a hierarchical layered structure. This architecture allows for strong interaction between polymer gel matrix and adjacent nanocellulose, which can contribute to the enhancement of mechanical properties [11,12,13]. On account of the introduction of -OH groups on polymer chains, poly (vinyl alcohol) (PVA) is capable of forming hydrogels with crosslinking, which exhibit intrinsic hydrophilicity, biocompatibility, biodegradability, high crystallinity and the feasibility of blending with nanocellulose [14]. Because the –OH groups on the nanocellulose surface can construct a stable hydrogen bonding system with the –OH groups of the PVA molecular chains, nanocellulose is ideally suitable for reinforcing nanofillers for PVA hydrogels. Because of their higher aspect ratio, larger size, chain entanglement and the overlap of the elongated nanofibers, cellulose nanofibers (CNFs) can form a more stable and stronger 3D network microstructure over cellulose nanocrystals (CNCs), providing a more significant reinforcing effect on PVA-based composite hydrogels [15].

In order to further endow the hydrogels with electro-conductivity, conductive hydrogels are prepared by introducing conductive materials, including conducting polymers, metallic nanomaterials and carbon nanoparticles [16,17,18,19] into hydrogels through a crosslinking approach [20]. Generally, the integration of these conductive materials can also result in the promotion of the toughness and even viscoelasticity of hydrogels due to the nanocomposite-reinforced mechanism [21]. As one of the most promising nanomaterials, graphene (GN) is a monolayer of two-dimensional carbon materials organized laterally in a honeycomb-like lattice [22]. In recent years, people have attempted to increase the mechanical strength and electrical conductivity of composite hydrogels by incorporating conductive nanomaterials. For example, Cai et al. demonstrated a type of CNTs/PVA hybrid hydrogels towards stretchable sensors [23]. Tong et al. found that the introduction of a certain amount of CNTs into the PVA hydrogel could improve the mechanical property and stretchability to some extent [24]. Nevertheless, because GN is very difficult to disperse well in aqueous suspension or hydrogel matrix, the non-homogeneity resulting from GN aggregation has adverse effects on the formation of the gel network, toughness and electro-conductibility for the composite gels, which is still an unsolved issue. Fortunately, CNFs can not only enhance the hydrogel matrix, but also effectively disperse the dispersion of GN in water. Therefore, we believe that the incorporation of nanocellulose can effectively solve this issue.

In addition, self-healing materials have attracted increasing attention. Self-healing hydrogels with reliability, durability and safety are capable of re-establishing their integrity, properties and gel network structure after damage, which can significantly extend the service life of hydrogel-based electronics [11,25]. Self-healing hydrogels are usually achieved by constructing dynamically reversible chemical bonds, such as hydrogen bonds, host–guest interactions and hydrophobic interactions [26,27]. Haick et al. prepared a type of flexible and self-repairing sensing material fabricated via dispersing metal particles in polyurethane diol towards a self-healable electrode [28]. Park et al. developed a class of electroconductive hydrogels with self-healing behavior via a polymerization of pyrrole monomers in agarose solution [25]. Nonetheless, most of the previously reported self-healing electronics suffer from poor stretchability, which can hardly be stretched over 100%. Consequently, it is highly desirable to explore an ECHs platform which can ideally combine self-healing ability, high stretchability and electro-conductibility all together simultaneously.

Herein, we successfully fabricated a type of intrinsic self-healing, highly conductive and stretchable hydrogels towards flexible strain sensors. Specifically, CNFs and GN were introduced to form GN-CNF nanocomplexes, which achieved the homogeneous distribution of GN in the PVA gel network. The incorporation of GN-CNF nanocomplexes considerably improved the electro-conductibility, elasticity and mechanical strength for the GN-CNF@PVA hydrogels. The hydrogen bonding system, entangling of PVA and GN-CNF, and dynamic and reversible multi-complexation that resulted from borax cross-linking contributed to the construction of the combined reinforcing and conductive network within the composite hydrogels. The strain sensors assembled by the GN-CNF@PVA hydrogel could sense and monitor the real-time body motion, demonstrating potential application in wearable sensing devices.

## 2. Materials and Methods

### 2.1. Materials

Sulfuric acid (98 wt%, Nanjing Chemical Reagent) was adjusted to 48 wt% concentration and cellulose powder was dried for 12 h at 50 °C before use. Sodium tetraborate decahydrate (borax, ≥99.5%) and polyvinyl alcohol (PVA, 99% hydrolyzed, *M*_w_ = 146,000~186,000 g mol^−1^) and were obtained from Sigma Chemical. Graphene (GN) was provided by Nanjing Kefu Nano-Tech Co. Ltd (Nanjing, China).

### 2.2. Isolation of Cellulose Nanofibers (CNFs) and Synthesis of Graphene-CNF/Poly (Vinyl Alcohol) (GN-CNF@PVA) Hybrid Hydrogels

In our previous work, cellulose nanofibers (CNFs) were isolated from cellulose powder by a combined acid hydrolysis and ultrasonification treatment [29,30]. The cellulose powder was hydrolyzed by using 48% by weight of sulfuric acid, wherein the ratio of acid to fibers was 20:1. The cellulose/acid solution was vigorously agitated at 45 °C for 1 h, and then the reaction was stopped by diluting 15-fold with cold water. The mixture was filtered under vacuum several times and the slurry obtained was re-dispersed in water. The excess acid was removed by centrifugation at room temperature for 25 min at 12,000 rpm, then the water was dialyzed in a dialysis tube with a molecular weight of 12,000–14,000 to pH = 7. To further improve the dispersibility, the CNFs aqueous suspension was sonicated in a 0.8 kW ultrasonic cell disrupter for 150 min to separate CNF at 0 °C. Finally, the concentration of CNF suspension was controlled at 1.0 wt%.

The GN-CNF@PVA hybrid hydrogels were prepared as follows. Firstly, the different amounts of graphene (GN) were slowly added into 200 g of CNF dispersion (1.0 wt%, containing 2 g of CNF) to form suspension, which was then stirred and sonicated at 300 W for 15 min at room temperature. The aqueous suspension containing GN-CNF nanocomplexes was evaporated to a weight of 80 g in a rotary evaporator. Specifically, the weight proportion of GN to CNFs were fixed at 0.15:1, 0.25:1 and 0.35:1, and the GN-CNF counterparts were designated as GN-CNF-A, GN-CNF-B and GN-CNF-C nanocomplexes, respectively. Meanwhile, 2.0 g of polyvinyl alcohol (PVA) was dissolved and stirred in 20 mL of deionized water at 90 °C. After a complete dissolution of PVA, the dissolved PVA solution was slowly added to 80 g of GN-CNF aqueous suspension, and stirring was continued for 0.5 h at 90 °C. Finally, 0.4 g of sodium tetraborate decahydrate was dispersed into the mixture under agitation. After the borax was completely dissolved, the reaction system was cooled to room temperature to form GN-CNF@PVA hybrid hydrogels. The hydrogels with the weight proportion (GN to CNFs) of 0.15:1, 0.25:1 and 0.35:1 were designated as GN-CNF@PVA-A, GN-CNF@PVA-B and GN-CNF@PVA-C, respectively (Table 1). As a reference, pure PVA hydrogels and CNF-PVA hydrogels were synthesized via the same procedure in the absence of CNFs or GN.

### 2.3. Characterizations

Hydrogel densities (*ρ*, g cm^−3^) were calculated from the weight and size of the specimens. Hydrogel specimens (initial weight = *W*_i_) were dried at 45 °C to a steady weight (*W*_d_). The moisture content (*W*_c_) was obtained from Equation (1):

(1) $$W_{c}=\frac{W_{i}-W_{d}}{W_{i}}\times 100\%$$

The morphology of CNFs, GN and GN-CNF dispersions were analyzed on a transmission electron microscope (TEM, JEM-1400, JEOL, Tokyo, Japan) at 80 kV. The gel morphology was investigated by a scanning electron microscope (SEM, JSM-7600F, Tokyo, Japan) at 5.0 kV. Fourier transform infrared (FTIR) analysis was conducted using a spectrophotometer (Thermo Fisher, San Jose, CA, USA) at a 4 cm^−1^ resolution from 4000 to 600 cm^−1^. X-ray diffraction (XRD) analysis of GN, CNFs, PVA and GN-CNF@PVA was performed on an X-ray diffractometer (Ultima IV, Japan) at 40.0 kV and 2*θ* = 5~40°. The rheological viscoelastic parameters, such as loss modulus (*G*″) and storage modulus (*G*′) versus *ω* were tested through the angular frequency (*ω* = 0.1 to 100 rad s^−1^) at *γ* = 1% at room temperature. The complex modulus (G*) were approached by Equation (2).

(2) $$G^{*}=\sqrt{G^{\prime 2}+G^{\prime \prime 2}}$$

Compression tests were carried out on the hydrogel cylinders (height~10.0 mm and diameter~35.0 mm) by a test machine (SANS CMT4304, Sans Testing Machine, Shen Zhen, China) at 10.0 mm min^−1^ compression speed. The uniaxial tensile measurements were conducted on the square specimens (50 × 5 × 5 mm^3^) with a stretching rate of 20.0 mm min^−1^, and the original distance between two clamps (*L*_0_) was fixed at 20.0 mm. The tensile stress (*σ*_t_) and compressive stress (*σ*_e_) were approached by dividing the force (*F*) by the original cross-sectional area (*A*_0_) of samples, while tensile strain (*ε_t_*) was obtained from the ratio of length (*L*) to the original length (*L*_0_). The elastic modulus (*E*) of the hydrogels was defined as the slope of the tensile stress-strain curve at 10–15% strain. The *σ* and *ε* values were calculated by Equations (3) and (4), respectively:

(3) $$\sigma_{t,e}\;=\;F/A_{0}$$

(4) $$\varepsilon_{t}\;=\;\left(L\;-L_{0}\right)/L_{0}\times 100\%$$

The conductivity measurements were performed on a CHI760E electrochemical workstation (Chenhua, Shanghai). Specifically, the gel specimens (1.0 × 0.5 × 0.5 cm^3^) were contained between two Pt electrodes. The hydrogel resistance was by Equation (5):

(5) R=U⁄I

where *R*, *U* and *I* were the hydrogel resistance (Ω), the potential (V) and the current of open circuit (A), respectively.

The conductivities of the gel samples were approached by Equation (6):

(6) $$\sigma =\frac{1}{R}\;\frac{L}{S}$$

where *L* was the thickness of hydrogel samples (cm), *S* was the area of hydrogel samples (cm^2^) and *R* was the resistance of hydrogel samples (Ω).

To assess the self-healing property, square gel specimens were first cut into parts, which were immediately brought into contact in air without stimulation. After a certain time of the healing process, the tensile tests were performed to assess the self-healing performance. The healing efficiency of the elongation at break (*ƒ*) was calculated from the maximum elongation at break ratio of healed hydrogels (*ε*′) to the original ones (*ε*) by Equation (7), where *ε*′ and *ε* were defined as dividing the original length by the elongation of the healed and original hydrogels during stretching, respectively.

(7) ƒ=ε′/ε×100%

The strain-sensing performance of hydrogel-based sensors were evaluated using an electrochemical workstation at 8.0 V. A homemade stretching device was used to stretch the sensors (40 mm in length and 4 mm in diameter) to a certain strain.

## 3. Results and Discussion

### 3.1. Fabrication Process of GN-CNF@PVA Composite Hydrogels

**Figure 1:**
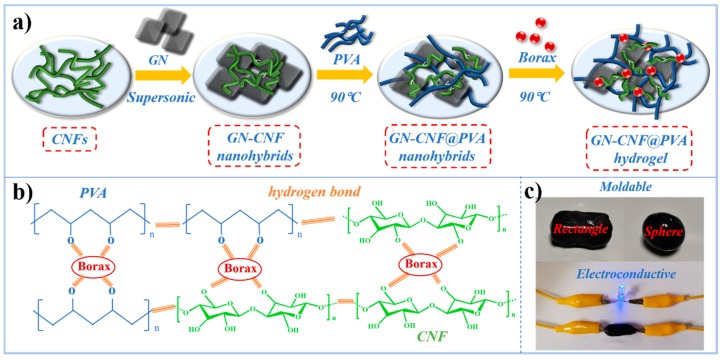
(**a**) Preparation process of graphene-cellulose nanofiber/poly (vinyl alcohol) (GN-CNF@PVA) hydrogels; (**b**) synthesis mechanism of the hydrogel network; (**c**) demonstration of the malleability and electro-conductivity of hydrogels.

The fabrication process of the GN-CNF@PVA hydrogel is presented in Figure 1a. The homogeneous CNF dispersion was extracted by a combined hydrolysis and sonication treatment. Because of their ideal dispersibility and small size, CNFs were used as an appropriate nanocarrier for promoting the dispersion of GN in aqueous medium, forming the interconnected conductive pathways. Subsequently, in the absence of any surfactant, the homogeneously dispersed GN-CNF nanocomplexes were distributed into the PVA polymer network to construct the GN-CNF@PVA hydrogel with 3D conducting pathways. During the gelation process, borax was quickly decomposed into B(OH)_4_^−^ and B(OH)_3_ after being dissolved in water (Figure 1b). Among them, the multi-complexation occurred through the attachment of borax to neighboring –OH groups of the PVA and CNFs [24]. The tetrahedral B(OH)_4_^−^ ion with four -OH groups at each corner could be reversibly complexed with cis-diol sites of GN-CNF nanocomplexes in water and PVA chains. Consequently, with the presence of borax cross-linker, GN-CNF nanocomplexes and PVA chains could be dynamically and reversibly complexed together. The multi-complexation, hydrogen bonding system and the entanglement of polymer chains successfully established a hierarchical 3D hydrogel network. As shown in Figure 1c, the GN-CNF@PVA gels obtained with a high water content (~95%) and a small density (~1.5 g cm^−3^) exhibited a smooth surface. These free-standing hydrogels with an electro-conductivity (~3.55 S m^−1^) could be readily molded into various shapes.

### 3.2. Morphology of GN-CNF Nanocomplexes

**Figure 2:**
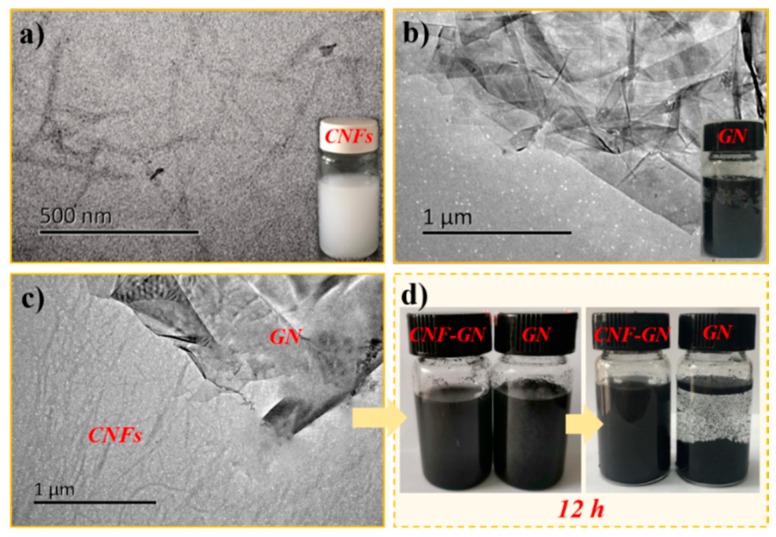
Transmission electron microscope (TEM) images of CNFs (**a**), GN (**b**), and GN-CNF nanocomplexes (**c**); (**d**) macroscopic pictures of pure GN and GN-CNF dispersions after a 12 h resting.

As shown in the TEM images of Figure 2a, CNFs with a high aspect ratio exhibited an average diameter and length of 28 ± 8 nm and 653 ± 73 nm, respectively, forming a homogeneous colloid in water. GN exhibited a typical lamellar structure and agglomerated overlap state in water mainly because of the intermolecular stacking attraction and van der Waals interactions (Figure 2b), indicating the poor dispersity of pure GN in aqueous medium [31]. The morphology characteristics of GN-CNF nanocomplexes can be clearly observed in Figure 2c. Obviously, the GN aggregates were divided into thinner GN layers without significant overlap in the large area. Because of the electrostatic repulsion behavior of CNFs, the hydrophobic interactions between specific crystalline surfaces on the CNFs and GN, CNFs were capable of uniformly dispersing GN in water [32,33]. As shown in Figure 2d, the pure GN and GN-CNF nanocomplexes were dispersed well after 1 h of stirring. However, after 12 h, due to the inherent intermolecular interactions, the pure GN formed a large-scale aggregation and precipitated in water, while the GN-CNF nanocomplexes remained homogeneously dispersed in water, further demonstrating the dispersing ability of CNFs for GN in water.

### 3.3. Chemical Structure of GN-CNF@PVA Composite Hydrogels

**Figure 3:**
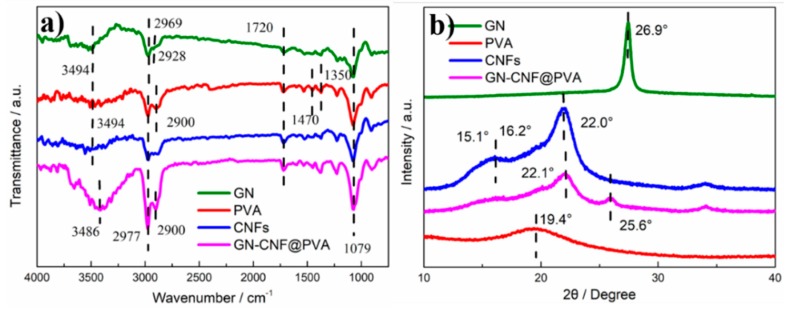
(**a**) Fourier transform infrared (FTIR) analysis and (**b**) X-ray diffraction (XRD) results of GN, CNFs, PVA hydrogel and GN-CNF@PVA hydrogel.

Figure 3a displays the FTIR curves of GN, CNFs, PVA hydrogel and GN-CNF@PVA hydrogel. Due to the defects of graphitic structure, the absorptions of CH_2_ and CH_3_ groups occurred at 2928 and 2969 cm^−1^, respectively [34]. CNFs showed representative peaks at 1079, 1720, 2977 and 3494 cm^−1^, due to C–H bending, -CH_2_ in-plane bending, C–H stretching and O–H stretching vibration, respectively [35]. For the pure PVA hydrogel, the absorptions at 1350 and 1470 cm^–1^ corresponding to the B–O–C (stretching relaxation) verified the crosslinked network of borax and PVA [36]. When the GN-CNF nanocomplexes were distributed into PVA network, most featured peaks of cellulose overlapped with others. The absorption appeared at 2900 cm^−1^ due to the C–H symmetric stretching of methylene group [37]. Compared to the pure PVA gel, the featured absorptions of the hydrogen bonds at 3494 cm^−1^ and 3486 cm^−1^ moved from the higher wave zone to the lower wave zone, revealing the strong linkage between the PVA and the GN-CNF nanocomplexes [38]. As shown in the XRD patterns of Figure 3b, the pure GN had a sharp diffraction peak at 2θ = 26.5° [39]. CNFs presented a prominent peak at 2θ = 22.0° and a lower overlapped peak at 2θ = 15.1° and 16.2°, corresponding to (101) and (002) planes [29]. PVA hydrogel exhibited a diffraction peak at 2θ = 19.4° [40]. For the GN-CNF@PVA hydrogel, the characteristic peak of PVA vanished, and the featured peaks of GN and CNFs were slightly shifted to 2θ = 25.6° and 22.1°. These results illustrated the dynamic interaction between borate ions, PVA and GN-CNF nanocomplexes [41].

### 3.4. Rheological Behavior of Composite Hydrogels

**Figure 4:**
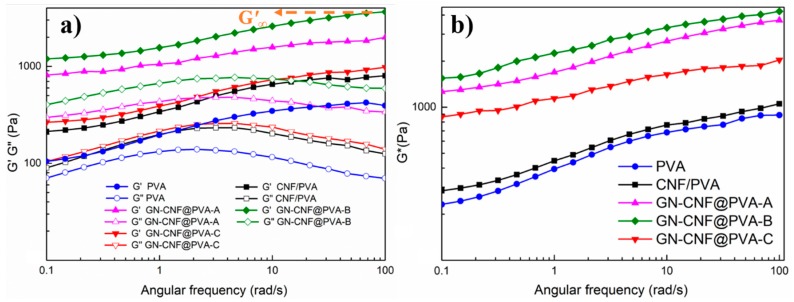
Rheological behavior of different gels under room temperature. (**a**) G′ and G″ versus ω; (**b**) frequency dependence of G* versus ω.

The dynamic oscillatory frequency sweep measurements of all hydrogels were tested at ω = 0.1~100 rad s^−1^ (Figure 4a). Within the entire frequency span, G″ were generally lower than G′, revealing the hydrogels had a fairly stable and strong permanent network [42]. The hydrogel viscoelasticity was improved with the integration of GN-CNF nanocomplexes. Compared with pure PVA and CNF/PVA gels, the G′_∞_ (high-frequency plateau of G’) value of GN-CNF@PVA-B hydrogel was increased by almost 5-fold and 9-fold, respectively. Among all hydrogels, the GN-CNF@PVA-B had the highest G′ (~3700.5 Pa) and G″ (~770.4 Pa) values (Table 2). This indicated strong interaction and entanglement between the PVA chain and the GN-CNF nanocomposite, as well as a more stable and higher crosslink [38]. When the GN content reached 0.7 wt%, the excessive GN affected the cross-linking degree at polymer intersections and hindered the creation of the hydrogel network, leading to the decrease of viscoelasticity. Figure 4b provided a more significant contrast of all hydrogels, where GN-CNF@PVA-B hydrogel possess the highest G* (~4227.8 Pa) within the whole ω range.

### 3.5. Mechanical Properties of GN-CNF@PVA Hybrid Hydrogels

**Figure 5:**
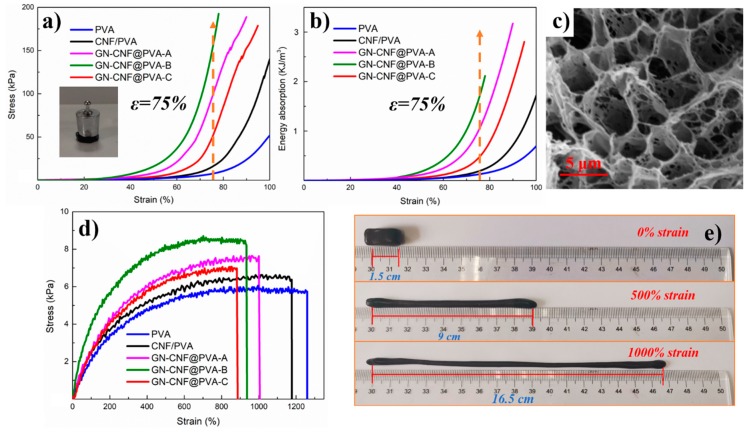
(**a**) Compression and (**b**) energy absorption graphs of various gels; (**c**) scanning electron microscope (SEM) image of GN-CNF@PVA-B hydrogel; (**d**) tensile graphs of PVA, CNF/PVA and GN-CNF@PVA gels; (**e**) demonstration of the stretchability of the GN-CNF@PVA-A gel.

The mechanical strength of hydrogels were comparatively evaluated by the energy absorption (E_a_) and the compressive stress (σ_e_) at ε = 75% (Figure 5a,b). Overall, GN-CNF@PVA-B hydrogels sustained higher stress than the other hydrogels. Among them, the σ_e_ values of CNF/PVA hydrogel (~15.3 ± 1.2 KPa) was about 2-fold higher than pure PVA gel (~7.4 ± 0.7 KPa), indicating that CNFs considerably improved the mechanical toughness of hydrogels. The homogeneously distributed GN-CNF nanocomplexes in a borax-crosslinked PVA gel network contributed to transferring the stress from polymer chains to GN-CNF nanocomplexes and halting the development of microcracks, thus resulting in the promotion in the hydrogel strength [43]. The σ_e_ values of GN-CNF@PVA-B was about 10 times greater than CNF/PVA hydrogels. One possible reason was that the B(OH)^4−^ ions were capable of reversibly complexing with the cis-diol sites of the GN-CNF nanocomplexes and PVA, and the entangling of the CNFs and PVA chains could also promote the establishment of the 3D gel network with a stronger structure [36]. In addition, when the content of GN was 0.7 wt%, the σ_e_ values of GN-CNF@PVA-C hydrogel was lower than the other hydrogels, indicating that the excess GN could reduce the effective crosslinking of GN-CNF nanocomplexes and PVA matrix. The energy absorption-strain curves displayed a more obvious contrast of all the gels in Figure 5b. The GN-CNF@PVA-B hydrogel reached an E_a_ value of 1.8 kJ m^−3^ at the 75% strain level (Table 3), which further demonstrated that GN-CNF nanocomplexes could reinforce compressive performance of hydrogels. As displayed in the SEM of Figure 5c, the porous structure of GN-CNF@PVA hydrogel confirmed the 3D interconnected network, and the GN-CNF nanocomplexes was uniformly dispersed in the hydrogel, which contributed to the enhancement of mechanical strength.

The tensile stress-strain graphs for different hydrogel samples are presented in Figure 5d. The incorporation of GN-CNF nanocomplexes considerably enhanced the tensile stress (σ_t_) of the gels. Pure PVA hydrogel showed the lowest σ_t_ value (~5.9 ± 0.3 KPa), while the GN-CNF@PVA-B hydrogel (~8.5 ± 0.4 KPa) had the highest σ_t_ value. As the content of GN-CNF nanocomplexes rose, the σ_t_ were gradually enlarged, while the elongation at break (ε_t_) were gradually declined. The ε_t_ of the original PVA hydrogel reached 1264.3 ± 50.2%, but the GN-CNF@PVA-B hydrogel decreased to 936.7 ± 23.5%. The well-dispersed GN-CNF nanocomplexes could interact with the PVA matrix, forming plenty of cross-linking points with PVA. Furthermore, the hydrogel network could dynamically break and recombine owing to borax, and thus GN-CNF nanocomplexes could act as effective reinforcement to improve the hydrogel strength [44]. The decrease of elongation at break resulted from the increased interaction between PVA chains and GN-CNF nanocomplexes, which hindered the migration of the PVA chains [45]. When the GN content was above 0.5 wt%, both the σ_t_ and the ε_t_ values were decreased. This result indicated that the addition of excess GN caused the formation of aggregates and denser polymer network, which had a severe adverse effect on the toughness and stretchability of the hydrogel [39]. In addition, the elongation at yield strength and yield strength were also two important indexes for hydrogels in tensile properties. As shown in Table S1, GN-CNF@PVA-B hydrogel demonstrated the highest yield strength (~8.5 kPa), which was consistent with the above results. Overall, GN-CNF@PVA-B hydrogel had the largest tensile properties. The high tensile properties of the hydrogel are more intuitively demonstrated with a tensile deformation of about 1000%, as shown in Figure 5e. Although these hydrogels exhibited good tensile properties, there were still stretchability limitations for their application in wearable e-skin devices due to the plastic deforming at low strains.

### 3.6. Self-Healing Properties of GN-CNF@PVA Hybrid Hydrogels

**Figure 6:**
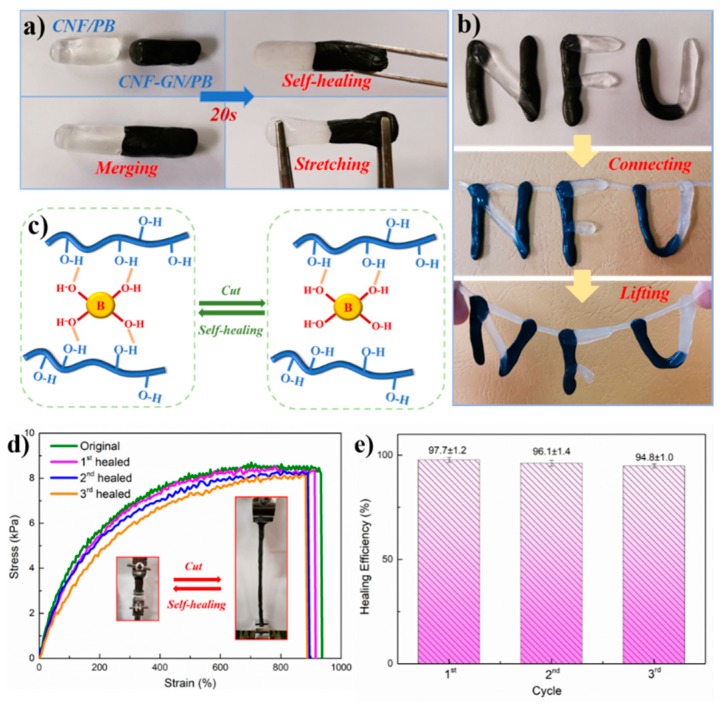
(**a**) The self-healing behavior of merging GN-CNF@PVA and CNF/PVA gels together; (**b**) photographs of the excellent healing ability in the different words for GN-CNF@PVA and CNF/PVA hydrogels; (**c**) self-healing mechanism of GN-CNF@PVA hydrogel; (**d**) stress-strain plots of original and self-healed GN-CNF@PVA-B hydrogels; (**e**) the values of healing efficiency in elongation at break after multiple breaking/healing cycles.

Figure 6 demonstrates the self-healing performance of these gels. Two freshly prepared GN-CNF@PVA and CNF/PVA hydrogels were put together (Figure 6a). After contact for 20 s, their surfaces were merged together and autonomously self-healed to a monolithic hydrogel without stimulation, revealing a rapid and intrinsic self-restore capability. The self-healed hydrogels could even be stretched without damage at the contacting interface. The moldable hydrogels with “NFU” shape still exhibited excellent self-healing ability when being connected and even lifted (Figure 6b). The ideal self-healing behavior with GN-CNF@PVA hydrogels resulted from the hydrogen bonding system, the dynamic and reversible multi-complexation between -OH groups of GN-CNF, PVA and borax [38]. The self-healing mechanism of hydrogels is shown in Figure 6c. These hydrogen bonds could be readily damaged and re-established, and thus the hydrogel could be remoulded and reformed repeatedly, endowing the hydrogels with intrinsic self-repairing ability and malleability at room temperature [24]. To explore the repeatability of in situ self-healing behavior, the GN-CNF@PVA-B hydrogels were circularly split at identical locations and repeatedly self-healed under constant conditions three times. Figure 6d presents the stress-strain plots of the original and restored hydrogels after various cutting and healing loops, and the corresponding healing efficiencies are presented in Figure 6e. The 1st, 2nd and 3rd healing efficiency reached up to 97.7 ± 1.2%, 96.1 ± 1.4%, and 94.8 ± 1.0%, respectively. The high healing efficiency was ascribed to the excellent healing repeatability of GN-CNF@PVA-B hydrogels. Nevertheless, a slight decline in self-healing efficiency value could not be avoided, because a small number of covalent bonds in neighboring polymer chains were permanently destroyed during the damage [46].

### 3.7. Electrochemical Performance of Hydrogels

**Figure 7:**
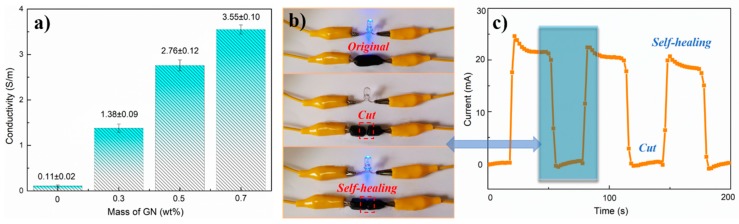
(**a**) Conductivity variation of GN-CNF@PVA gels with various amount of GN; (**b**) self-healing ability of conducting network in GN-CNF@PVA hydrogel when connected into a circuit; (**c**) current changes of the GN-CNF@PVA-B hydrogel during multiple cutting/healing cycles.

Figure 7a shows the electro-conductivity change of GN-CNF@PVA with various content of GN. The electrical conductivities for all hydrogels presented a non-linear rise as the GN amount increased. At GN = 0.7 wt%, an electro-conductibility of 3.55 S·m^−1^ was generated for GN-CNF@PVA. As reported previously, CNFs could endow pathways for ion and electron transporting with their electrochemical hybrids with other materials [47]. Consequently, the 1 d configuration of CNFs allowed less percolation concentration of GN-CNF nanocomplexes to accomplish a continued conducting network, and the homogeneously dispersed GN-CNF nanocomplexes additionally created efficient conducting paths inside GN-CNF@PVA hydrogels [35]. Besides, the multi-complexation among borate ions, PVA and GN-CNF nanocomplexes were capable of providing the hydrogels with conducting paths, thus facilitating the electronic transmission [48]. To clearly demonstrate the self-restoring capability of the conducting network inside hydrogels, GN-CNF@PVA hydrogel was connected to a closed circuit (Figure 7b). After the circuit was connected, the light-emitting diode (LED) bulb was immediately lit, suggesting the ideal electro-conductibility of the GN-CNF@PVA hydrogel with a hierarchical conducting network [46]. When the GN-CNF@PVA hydrogel was completely cut into two pieces, the blue bulb was extinguished under an open circuit. Once the two pieces self-healed, the LED indicator could be turned on again. Figure 7c displays the current variations over time for GN-CNF@PVA-B hydrogel during damaging and self-healing cycles. The electrical current of the self-healed hydrogel remained relatively stable under multiple cutting/healing cycles. The demonstration illustrated that the GN-CNF@PVA hydrogel had an ideal electrical self-healing capacity, suggesting many promising applications in the field of self-repairing soft electronics [24].

### 3.8. Strain-Sensitive Performance of GN-CNF@PVA Hydrogel-Based Sensor

**Figure 8:**
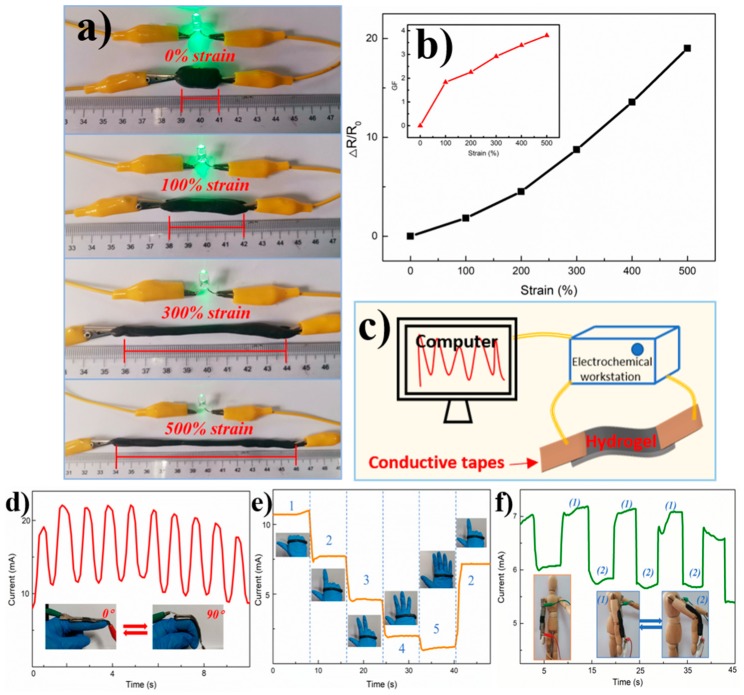
(**a**) Demonstration of the variations for light-emitting diode (LED) illumination vs different strain of the GN-CNF@PVA gels in a closed circuit; (**b**) the corresponding resistance variations due to the strain; (**c**) schematic illustration of strain sensor: current change of the GN-CNF@PVA hydrogels responding to rapidly finger bending (**d**) and various hand gestures (**e**); (**f**) the recorded current variations of the GN-CNF@PVA hydrogel-based strain sensor in arm bending.

To assess the practicability of GN-CNF@PVA gel for use in wearable sensing applications, the GN-CNF@PVA hydrogel embedded in a closed circuit equipped with a green bulb (Figure 8a). The illumination of the green bulb gradually deceased with the increasing applied strain (0%~500%), suggesting that the hydrogel electro-conductivity was highly sensitive to the applied strain [2]. As the applied tensile stress enhanced, the spaces between the conducting particles inside the hydrogel were enlarged, resulting in the increased resistance of hydrogels. This feature allowed these hydrogels to be used in the field of motion sensing. The strain sensitivity of the hydrogel-based sensors could be assessed quantitatively by the gauge factor (GF), calculated from the equation, GF = ((R−R_0_)/R_0_)/ε, where ε was the strain, R and R_0_ were the resistance when stretching and the original resistance, respectively. From Figure 8b, it was found that GF values increased as the applied strain increased. When the strain increased to 500%, the value of GF reached up to 3.8, which was superior to various hydrogel-based strain sensors previously reported, such as the k-carrageenan/Polyacrylamide double-network hydrogels (GF~0.3 at 500% strain) [49] and PVA-PVP hybrid hydrogels (GF~0.48 at 200% strain) [2]. In general, the GF values of hydrogel-based strain sensors increased with the increasing strain. Under a high strain level, the GF values of CNTs/PVA hydrogels [26] and Polyacrylamide-Polydopamine hydrogels [50] were 0.24 and 0.70 at 1000% strain, respectively, which were even lower than the GF value of GN-CNF@PVA hydrogels at 500% strain. Such an ideal sensitivity endowed GN-CNF@PVA hydrogels with great potential in strain sensors. To demonstrate a real application of the GN-CNF@PVA hydrogel-based strain sensor, the sensor assembled by hydrogels was installed on a human index finger using conducting tapes, and then two cables were connected to an electrochemical workstation to monitor the variation of current (Figure 8c). When the finger was rapidly bent from 0° to 90°, the current reduced gradually in response to the movements of the finger (Figure 8d), and the intensity variation of the current peak could accurately reflect the bending angle in real time [25]. Simultaneously, the displayed current variations exhibited practically constant peaks and shapes, indicating the repeatability, stability and reliability of the GN-CNF@PVA hydrogel-based strain sensors [51]. As shown in Figure 8e, more complicated hand motions could also be sensed easily by the assembled sensors. Each gesture for “one”, “two”, “three”, “four” and “five” generated a different current response, and the current response was completely restorable. Interestingly, the current response remained steady under a certain gesture. Therefore, the current variation was capable of monitoring various hand gestures precisely in real time [14]. Once again, the GN-CNF@PVA hydrogel-based strain sensor had a remarkable electrical stability and repeatably in human-motion detection.

The hydrogel-based strain sensor could further be attached onto a puppet to monitor the arm and leg movements (Figure 8f and Figure S1). During the arm and leg motions, the strain sensors were stretched to generate a tensile stress, which could destroy the conducting network and thus decrease the current values. More importantly, the bending and relaxation of the arms and legs could be easily distinguished based on the output current. The response behavior was repeatable, producing a similar current response by repeating the same motions [51,52]. Besides large-scale action, the hydrogel-based sensors could even detect some complex and unique waveforms as illustrated in Figure S1. For example, when handwriting the simple word “OK” or the shape of love on paper with the strain sensor, it was completely different from the response of current in the previously simple bending motion. It could be observed that the different motions exhibited different responses of current, resulting in the different waveforms. More importantly, when repetitive motion was applied, approximately constant response curves were produced, revealing an ideal reproducibility of these hydrogel-based strain sensors [53]. Therefore, the hydrogel-based strain sensor could not only distinguish simple human motions, but also identify complex spatial movements, which is beneficial to the further development of sign language recognition and communication of deaf-mute people [51].

GF and sensitivity were two important factors for hydrogel-based strain sensor. Many methods had been proposed to improve the GF values of strain sensors. For example, microcracks were introduced into the strain sensor at low strain, and the GF value could be increased quickly [54]. However, the low detection range of these strain sensors limited their application greatly. Then it was proposed to build a 3D conductive network and prepare a strain sensor with rough microstructure [55,56]. Even though the GF value was improved, the application of sensors was limited because it was difficult to completely contact with the skin. Therefore, fabrication of an ultra-thin sensor might be a promising way to increase GF values and sensitivity [57].

## 4. Conclusions

In conclusion, the current study prepared a novel type of highly stretchable, conductive and self-restorable hydrogel-based strain sensor, which was formed via dynamic and reversible multi-complexation between borax cross-linker, PVA matrix and GN-CNF nanocomplexes. Biomass-derived CNFs acts as templates and nanocarriers to support GN to create uniformly dispersed GN-CNF nanocomplexes in PVA gel matrix, enhancing the viscoelasticity, mechanical strength and electro-conductivity of the GN-CNF@PVA hydrogel. At an optimal content of 0.5 wt% GN, the GN-CNF@PVA-B hydrogel presented high stretchability (the elongation at break up to 1000%), high compressive strength (148.1 ± 10.4 kPa), excellent viscoelasticity (G’_max_ ≈ 3.7 kPa) and ideal electro-conductivity (up to 3.55 ± 0.1S m^−1^). Due to the dynamic reversible bonds formed by borate ions with GN-CNF nanocomplexes and PVA chains, the hydrogel exhibited rapid a self-healing behavior (within 20 s) with a high healing efficiency (97.7 ± 1.2%). Towards the practical wearable application, the strain sensor assembled by GN-CNF@PVA hydrogel was capable of effectively monitoring and distinguishing diverse human movements with a GF of about 3.8, demonstrating excellent sensitivity, repeatability and stability to the signals. Therefore, we expect the GN-CNF@PVA hydrogel-based strain sensor can attain promising applications in intelligent wearable electronics.

## Figures and Tables

**Table 1:** The ratio of substance in hydrogels.

Sample	GN	CNF	PVA
PVA	0.0%	0.0%	2.0%
CNF/PVA	0.0%	2.0%	2.0%
GN-CNF@PVA-A	0.3%	2.0%	2.0%
GN-CNF@PVA-B	0.5%	2.0%	2.0%
GN-CNF@PVA-C	0.7%	2.0%	2.0%

**Table 2:** Viscoelastic properties of hydrogels obtained from moduli plots.

Parameter	PVA	CNF/PVA	GN-CNF@PVA-A	GN-CNF@PVA-B	GN-CNF@PVA-C
High-frequency plateau of *G*′, *G*′_∞_(Pa)	421.1	790.1	1989.8	3700.5	973.3
Maximum *G*″, *G*″ (Pa)	141.3	237.6	471.3	770.4	252.9
Maximum G*, G* (Pa)	887.7	1051.0	3698.0	4227.8	2032.4

**Table 3:** Physical-mechanical performances of original gels.

Hydrogels	*σ_e_* at *ε* = 75% (KPa)	*E*_a_ at *ε* = 75% (kJ m^−3^)	*σ_t_* (KPa)	*ε_t_* (%)	*E* (KPa)	*W*_c_ (%)	*Ρ* (g cm^−3^)
PVA	7.4 ± 0.7	0.2 ± 0.1	5.9 ± 0.3	1264.3 ± 50.2	4.1 ± 0.5	96.3 ± 0.2	1.0 ± 0.1
CNF/PVA	15.3 ± 1.2	0.3 ± 0.1	6.6 ± 0.6	1177.7 ± 35.7	4.9 ± 0.8	95.5 ± 0.4	1.2 ± 0.3
GN-CNF@PVA-A	95.0 ± 8.3	1.0 ± 0.3	7.6 ± 0.5	1000.2 ± 36.8	6.3 ± 1.0	95.2 ± 0.1	1.3 ± 0.1
GN-CNF@PVA-B	148.1 ± 10.4	1.8 ± 0.4	8.5 ± 0.4	936.7 ± 23.5	9.4 ± 1.2	95.0 ± 0.2	1.5 ± 0.2
GN-CNF@PVA-C	48.0 ± 3.5	0.5 ± 0.2	7.1 ± 0.3	866.6 ± 28.4	5.2 ± 0.4	94.7 ± 0.3	1.6 ± 0.2

## Supplementary Materials

The following are available online at https://www.mdpi.com/2079-4991/9/7/937/s1. Figure S1: The recorded current variations of the GN-CNF@PVA hydrogel-based strain sensor in different motions: walking (a), handwriting the word “OK” (b), and handwriting the shape of love (c); Table S1: The yield index of original gels.
