# Supplementary material for: Highly Stretchable and Self-Healing Strain Sensors Based on Nanocellulose-Supported Graphene Dispersed in Electro-Conductive Hydrogels
Source: Nanomaterials (Basel). 2019 Jun 28;9(7):937. doi: 10.3390/nano9070937 (PMC6669678; doi:10.3390/nano9070937)
Supplement: Supplementary file 1 [file nanomaterials-09-00937-s001.pdf]

Supporting Information

Highly Stretchable and Self-Healing Strain Sensors Based on Nanocellulose-Supported Graphene Dispersed in Electro-Conductive Hydrogels

Chunxiao Zheng [1], Yiying Yue [2], Lu Gan [1], Xinwu Xu [1], Changtong Mei [1,*] and Jingquan Han [1,*]

1 College of Materials Science and Engineering, Nanjing Forestry University, Nanjing 210037, China; zhengcx0313@gmail.com (C.Z.); ganlu@njfu.edu.cn (L.G.); xucarpenter@aliyun.com (X.X.)
2 College of Biology and Environment, Nanjing Forestry University, Nanjing 210037, China; yue@njfu.edu.cn
* Correspondence: mei@njfu.edu.cn (C.M.); hjq@njfu.edu.cn (J.H.)

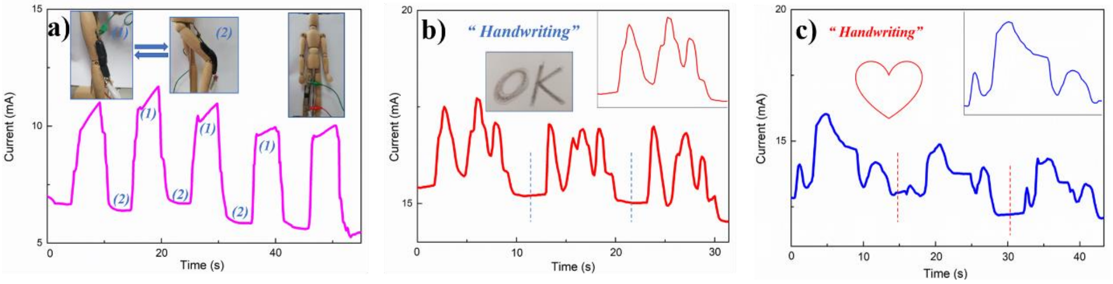

Figure S1. The recorded current variations of the GN-CNF@PVA hydrogel-based strain sensor in different motions: walking (a), handwriting the word "OK" (b), and handwriting the shape of love (c).

Table S1. The yield index of original gels

Parameter	PVA	CNF/PVA	GN-CNF@PVA-A	GN-CNF@PVA-B	GN-CNF@PVA-C
elongation at yield strength (%)	701.4 ± 25.6	688.3 ± 20.4	654.7 ± 14.6	606.5 ± 15.7	658.4 ± 17.3
yield strength (kPa)	5.7 ± 0.2	6.2 ± 0.3	7.3 ± 0.3	8.5 ± 0.4	6.7 ± 0.2
